# Plant epigenetics: from genotype to phenotype and back again

**DOI:** 10.1186/s13059-016-0920-5

**Published:** 2016-03-29

**Authors:** R. Keith Slotkin

**Affiliations:** Department of Molecular Genetics and Center for RNA Biology, The Ohio State University, Columbus, Ohio 43210 USA

## Abstract

A report on the *Plant Epigenetics: From Genotype to Phenotype* Keystone Symposium held in Taos, New Mexico, USA, 15–19 February, 2016.

## Introduction

In February 2016, the plant epigenetics community assembled in Taos, New Mexico, for the *Plant Epigenetics: From Genotype to Phenotype* Keystone Symposium. This meeting marked the latest contribution to the plant epigenetics field by the Epigenomics of Plants International Consortium, a US National Science Foundation-funded group that aims to disseminate plant epigenetics information to support and elevate this community of researchers. The conference began with a keynote address from Steven Jacobsen (University of California, Los Angeles), who provided an introduction and framework for the rest of the presenters to build from. Oral presentations ranged from established leaders in the field, junior faculty, and postdoctoral researchers, and the meeting included lively poster sessions filled with a wealth of unpublished data and associated models. Presentations ranged in organism, approach, and specific topic; however, seven strong themes stood out.

## Crop improvement

The most notable trend at this meeting was how, on many fronts, this field has recently deciphered complex epigenetic inheritance patterns at the molecular chromatin level, particularly for agricultural traits. For example, Robert Martienssen (Cold Spring Harbor Laboratory) identified the epiallele responsible for poor fruit production in the oil palm. His group generated an early assay to genotype palms before transplantation, saving valuable land resources for those trees that will produce high-quality fruit. Elizabeth Dennis (CSIRO, Australia) demonstrated that hybrid vigor acts on key metabolic pathways early in development to produce increased energy for *Arabidopsis* plants. She produced vigor mimics that are not hybrids, but have many of the same benefits. In addition to investigating the underlying mechanisms, several presentations focused on using epigenomic markers for agricultural improvement. Felix Seifert (University of Hamburg, Germany) discussed his research on maize heterosis, using small RNA distribution patterns to predict vigor relationships. Jon Reinders (DuPont Pioneer) used DNA methylation patterns as markers to predict key phenotypes in maize breeding programs.

## Stress and defense

A second notable trend was the focus on the epigenetic regulation of stress responses, shifting away from a *trans*-generational effect and toward epigenetic regulation within a single generation. Stress responses are being investigated at the single gene level and at the whole chromosome level with the formation and disassociation of heterochromatic chromocenters. Ortrun Mittelsten Scheid (Gregor Mendel Institute, Austria) demonstrated chromocenter dissociation during high temperature stress, and the number of days required to reset the normal organization of heterochromatin. Similarly, Fredy Barneche (Institut de Biologie de l’École Normale Supérieure, France) demonstrated light-induced chromocenter formation on de-etiolation, and the role of H1 linker histone variation in this process. Switching to plant defense (perhaps plant offense would be a more appropriate term), Claude Becker (Max Planck Institute for Developmental Biology, Germany) showed how certain plants produce and exude chemical compounds that are converted in the soil into histone deacetylase inhibitors. These compounds inhibit the growth of nearby competitor plants—a process called allelopathy. Claude determined which genes and pathways are perturbed in the affected plants.

One stress-related topic that received considerable attention at the meeting was priming, whereby a plant is more resistant to a stressor if it has previously encountered that same stress. Priming occurs within a single generation and constitutes an epigenetic memory that is being leveraged for crop improvement. Zoya Avramova (University of Nebraska) discussed the transcriptional memory of drought on particular stress response genes, while Anna Amtmann (University of Glasgow, UK) investigated osmotic and salt stress. She showed that the initial priming stress must occur at a specific young developmental stage, and discussed the histone deacetylation necessary for the priming response.

## Gene regulation

The epigenetic regulation of genes underlies the more complex inheritance patterns of traits involved in crop improvement and stress tolerance. Several presentations focused on the epigenetic regulation of single genes, which drive cellular memory of the environment or developmental timing. Caroline Dean’s lab (John Innes Centre, UK) discussed the memory of winter held at the chromatin level that is responsible for vernalization, the process by which prolonged exposure to cold temperatures promotes flowering. Her lab investigated the timing of flowering controlled by the *FLOWERING LOCUS C* (*FLC*) gene, and a long non-coding RNA produced from this same locus responsible for the regulation of *FLC*. They performed cutting-edge single-molecule fluorescence in situ hybridization that followed the pairing and expression dynamics of the key regulatory *FLC* locus through the prolonged response to cold required for vernalization. Ido Keren (SUNY-Stony Brook) researched a histone deubiquitinase that acts on *FLC* when the gene is activated, while the memory of cold treatment at *FLC* is governed by histone H3 trimethylation of lysine 27(H3K27me3). Iva Mozgová, a postdoctoral fellow with Lars Henning (Swedish University of Agricultural Sciences) researched the role of the polycomb repressive complex proteins that initiate the H3K27me3 mark in regulating somatic embryogenesis. Toshiro Ito (Temasek Life Sciences Laboratory, Singapore) investigated the timed induction of H3K27me3 in floral stem cells and in flower development. Both Toshiro and Doris Wagner (University of Pennsylvania) discussed the mechanism by which the chromatin modifiers that direct H3K27me3 are recruited or evicted from their target loci. They elegantly demonstrated that developmentally induced transcription factors control this recruitment and eviction.

The most perplexing epigenetic gene regulation is paramutation, whereby the repressed state of an allele can be transferred to an active allele. Maike Stam (University of Amsterdam, The Netherlands) discussed her research on paramutation at the maize *b1* locus, which is dependent on a series of seven tandem repeats far upstream of the *b1* coding region. Maike investigated the chromatin structure of the repeats and their regulation by proteins involved in RNA-directed DNA methylation (RdDM). Karen McGinnis (Florida State University) is studying the same RdDM mutants in maize; these produce striking phenotypes that she has connected to regulation by the phytohormone abscisic acid and altered nucleosome positioning. Mario Arteaga Vázquez (Universidad Veracruzana, Mexico) also spoke about paramutation at the *b1* locus, and then transitioned his research to examples of paramutation in *Marchantia*, a liverwort and rudimentary land plant that is an up-and-coming system for plant molecular biology.

## Nucleolar dominance

A major breakthrough discussed at the meeting was the distinction between active and inactive nucleolar organizing regions (NORs) within a single ecotype. When hybrids are produced, one set of NORs is dominant to the other set, creating a phenomenon called nucleolar dominance. Craig Pikaard’s laboratory (University of Indiana) mapped the ribosomal RNA variants within the *Arabidopsis* reference strain and found that, within this one strain (not a hybrid), one NOR is transcriptionally active while the other is silenced. Fernando Rabanal (Gregor Mendel Institute, Austria) came to a similar conclusion with a different quantitative trait locus-based deep sequencing approach. Together, Rabanal and Pikaard demonstrated that the entire NOR operates as a single unit that drives the hierarchical dominance relationships between NORs, a conceptual breakthrough in the nucleolar dominance field.

## Histone dynamics: modifications and variants

The epigenetic regulation of crop traits, stress response, and more broadly gene regulation manifest at the molecular level as changes in chromatin structure that can provide a memory of a transcriptional state. A major focal point of the presentations converged on how chromatin structures and domains are established and molecularly delineated, with particular emphasis on the associated histone dynamics that control chromatin structure. Steven Jacobsen discussed the establishment of histone H3 monomethylation of lysine 27 (H3K27me1) chromatin mark by the ATXR5 and ATXR6 proteins. Failure of ATXR5/6 to mark heterochromatin with H3K27me1 results in DNA overreplication. This activates DNA repair pathways that suppress the overreplication-induced DNA damage. He described his model of how overreplication in the *atxr5/6* mutants occurs due to a failure to resolve conflicts when both transcription and replication occur in heterochromatin. Scott Michaels (Indiana University) found nuclear repair pockets in the *atxr5/6* mutants responsible for repairing DNA breaks. He speculated that these pockets provide a sequestered chromosome repair microenvironment that provides access for repair proteins and excludes other chromatin modifiers.

Two presentations focused on the idea that certain histones and/or modifications are mutually exclusive (not on the same nucleosome). Fred Berger (Gregor Mendel Institute, Austria) focused on the histone variant H2A.W, which is plant-specific and concentrates at the pericentromere. He showed that mononucleosome isolation could identify mutually exclusive histone marks that divide the chromosome into distinct regulatory domains. In parallel, Crisanto Gutiérrez (Centro de Biología Molecular Severo Ochoa, Spain) used live fluorescent reporters to demonstrate that dividing cells have histone H3.1, but this is mutually exclusive with histone H3.3, which replaces H1.1 in the last cell cycle. Two other presentations centered on how histone variants are targeted to specific regions of the chromosome. Inna Lermontova (Institute of Plant Genetics, Germany) discussed a factor responsible for directing the centromeric histone H3 variant to the centromere core for assembly, and that this process operates in a cell cycle-specific manner. Paul Fransz (University of Amsterdam, The Netherlands) discussed the organization of nuclear chromosomal territories, the association of centromeric DNA with the nuclear envelope, and mutants that perturb this organization.

## DNA methylation dynamics

The DNA methylation mark in plants is transmitted from one generation to the next, propagating epigenetic information in a much more direct route compared to animals. Several presentations focused on the mechanism and evolutionary implications of cytosine DNA methylation. Robert Schmitz (University of Georgia) presented an evolutionary history of gene body methylation. Using data from a number of plant species, he focused on the evolutionary function of methylation within genes. Nathan Springer (University of Minnesota) discussed DNA methylation that accumulates in the CHH context (where H is any base but G) in the complex maize genome. Nathan demonstrated that most CHH methylation in maize is at islands that mark the boundary between heterochromatin and a neighboring gene. Along with DNA methylation, Xiaofeng Cao (Chinese Academy of Sciences) researched how the histone demethylase protein IBM1 functions to remove heterochromatic histone marks from genes and restrict this mark to transposable elements, thus providing a distinct boundary force to segregate gene chromatin states in complex genomes such as rice and maize.

A key topic addressed in many presentations focused on the evolutionary dynamics of DNA methylation. Nathan Springer presented data demonstrating that methylation within the maize genome is stability-inherited over long periods of time. Magnus Nordborg’s lab (Gregor Mendel Institute, Austria) investigated the natural variation of DNA methylation and its correlation with the environment. His research demonstrated that many epigenetic changes between ecotypes have a genetic (sequence) root cause, as single-nucleotide polymorphisms drive gene body methylation from a distance. Frank Johannes (Technical University Munich, Germany) demonstrated that the rate of spontaneous epimutations is highest in the CG sequence context, as these sites are not retargeted by RdDM. He proposed the interesting argument that CG epimutations can be viewed as a molecular clock. After methylation, Jonathan Cahn from Ryan Lister’s lab (University of Western Australia) identified methylation-binding proteins, with the hope of characterizing how the cell interprets the DNA methylation signal to dictate expression changes, while Jian-Kang Zhu (Purdue University/Shanghai Center for Plant Stress Biology) discussed the specific biochemical steps associated with the removal of DNA methylation via DNA glycosylase enzymes.

## Small RNA function and movement

The function of small RNAs was investigated in several presentations. Jixian Zhai from the Jacobsen laboratory (University of California, Los Angeles) described his recent sequencing capture of RNA polymerase IV (Pol IV) transcripts, which are cleaved into 24-nucleotide small RNAs that participate in RdDM. Jixian showed that the Pol IV transcripts are very short, and then presented his model proposing that each individual Pol IV transcript is cleaved into one small RNA. Andrzej Wierzbicki (University of Michigan) discussed his lab’s improved methods of identifying RNA polymerase V (Pol V) scaffolding transcripts. He showed that Pol V uses internal promoters and produces bidirectional short transcripts. Lastly, two presentations focused on the mobility of small RNAs. Mathew Lewsey from the Ecker Lab (Salk Institute for Biological Studies) demonstrated that Pol IV small RNA production does not need to occur in the same cell targeted by RdDM, as endogenous Pol IV small RNAs are mobile across graft junctions and target RdDM at many different transposable element regions in recipient tissues. In addition, R. Keith Slotkin (The Ohio State University) showed transposable element small RNA movement within the pollen grain from terminally differentiated pollen vegetative cells into the sperm cells, suggesting that small RNAs may function between one generation and the next.

## Conclusions

The topics presented at this meeting were highly variable in nature, ranging from the complex regulation of stress and heterosis to the precise mechanisms of RdDM. The subtitle of the meeting “from genotype to phenotype” was investigated at length, but the more complex route *from phenotype to genotype* was also explored, providing breakthroughs in the explanation of complex phenotypic phenomena, such as nucleolar dominance and stress priming. Working both from the bottom (mechanism) up and top (phenotype) down is a major strength of the plant epigenetics community, and this dual approach will enable high-impact science and, more importantly, continued breakthroughs on complex traits.

## Abbreviations

*FLC*: *FLOWERING LOCUS C*
H3K27me1: Monomethylation of histone H3 on lysine 27
H3K27me3: Trimethylation of histone H3 on lysine 27
NOR: Nucleolar organizing region
Pol IV: RNA polymerase IV
Pol V: RNA polymerase V
RdDM: RNA-directed DNA methylation

